# Using Lagrangian relaxation to locate hydrogen production facilities under uncertain demand: a case study from Norway

**DOI:** 10.1007/s10287-023-00445-3

**Published:** 2023-03-01

**Authors:** Šárka Štádlerová, Sanjay Dominik Jena, Peter Schütz

**Affiliations:** 1 Department of Industrial Economics and Technology Management, Norwegian University of Science and Technology, Trondheim, Norway; 2 School of Management, Université du Québec à Montréal, Montreal, Canada; 3 Interuniversity Research Centre on Enterprise Networks, Logistics and Transportation (CIRRELT), Montreal, Canada

**Keywords:** Multi-period facility location, Capacity expansion, Uncertain demand, Lagrangian relaxation

## Abstract

Hydrogen is considered a solution to decarbonize the transportation sector, an important step to meet the requirements of the Paris agreement. Even though hydrogen demand is expected to increase over the next years, the exact demand level over time remains a main source of uncertainty. We study the problem of where and when to locate hydrogen production plants to satisfy uncertain future customer demand. We formulate our problem as a two-stage stochastic multi-period facility location and capacity expansion problem. The first-stage decisions are related to the location and initial capacity of the production plants and have to be taken before customer demand is known. They involve selecting a modular capacity with a piecewise linear, convex short-term cost function for the chosen capacity level. In the second stage, decisions regarding capacity expansion and demand allocation are taken. Given the complexity of the formulation, we solve the problem using a Lagrangian decomposition heuristic. Our method is capable of finding solutions of sufficiently high quality within a few hours, even for instances too large for commercial solvers. We apply our model to a case from Norway and design the corresponding hydrogen infrastructure for the transportation sector.

## Introduction

According to the emission targets set in the Paris agreement, greenhouse gas emissions (as by 1990) must be decreased by $40 \%$ until 2030 (United Nations 2015). The Norwegian government has set even more ambitious goals regarding the emissions within the transportation sector (Regjeringen 2019). Specifically, the transition towards zero-emission fuels is a key step in order to meet these targets. The transition from fossil fuels towards hydrogen gained even more importance as countries with diversified energy carrier mix can better handle the current energy crisis in Europe (Crew 2022). IEA (2022) further states that the global energy crisis accelerates the urgency to use hydrogen, as it contributes to emission reduction targets as well as energy stability. With $92\%$ electricity produced from hydropower, Norway is well positioned to produce green hydrogen, which is required to be produced exclusively from renewable sources using electrolysis (EL).

In Norway, the sector of high-speed passenger ferries and car ferries is operated based on public contracts, and when renewing these contracts, hydrogen can be required as zero-emission fuel (Ocean Hyway Cluster 2020a). The demand from sectors that are operated based on public contracts may therefore be easier to predict and has a deterministic character as the transition can be forced based on the contracts. There are also alternative zero-emission energy carriers that are relevant in Norway, such as electric batteries and ammonia. However, the future market shares among these fuels are uncertain. Since demand from other relevant sectors such as land-based transport and the offshore sector is highly uncertain, having the ability to expand the production infrastructure is crucial to meet future demand (DNV GL 2019).

The above motivates our work on the real-world problem of locating hydrogen production facilities in Norway under uncertain demand. The decisions regarding opening location, time and capacity must be taken before the future demand is known. After uncertain demand is disclosed, decisions regarding capacity expansion and production, as well as demand allocation can be taken. The problem formulates as a large mixed-integer programming problem that is, in general, hard to solve. Specifically, as shown in Štádlerová et al. (2022), the problem can be solved with a commercial solver only for a few scenarios. In this paper, we, therefore, solve this problem using a solution method based on Lagrangian relaxation.

Our contributions are threefold. First, we provide a solution method based on Lagrangian relaxation for the multi-period facility location and capacity expansion problem under uncertainty that allows for solving problems with a sufficiently large number of scenarios within reasonable computing time. Our model formulation includes minimum production requirements motivated by the properties of the production technology for hydrogen. Such requirements can also be found in other industries, for example due to economic or technological considerations (such as minimum batch sizes). Still, our model formulation is general enough to also be applicable if such minimum production requirements do not exist. We compare the performance of the method to the one of Gurobi and discuss the quality of the Lagrangian bound. We further analyze the out-of-sample performance and discuss the value of the stochastic solution. Second, we study the hydrogen production infrastructure for different demand distributions and compare the first-stage solutions to the solution from the expected value problem. The computational results show that the Lagrangian relaxation provides tight lower bounds and that our solution method finds solutions of sufficiently high quality for all tested instances. We further analyze the value of the stochastic solution, indicating that for most problems, the solution of the expected value problem is of no practical use. Third, we analyze the solution obtained for the case of Norway, illustrating the practical usefulness and importance of our approach.

The remainder of this paper is structured as follows: The relevant literature is reviewed in Sect. 2. The mathematical model is introduced in Sect. 3. The solution method is detailed in Sect. 4. The case study is presented in Sect. 5 and the computational results are discussed in Sect. 6. Finally, we conclude in Sect. 7.

## Literature review

We split the literature review in two main parts. In Sect. 2.1, we provide a brief literature review on modelling deterministic and stochastic capacitated facility location problems with piecewise linear costs and/or capacity expansion. We also review facility location and supply chain design problems in the context of hydrogen infrastructure. Solution methods for facility location and supply chain design problems with a focus on two-stage stochastic problems are reviewed in Sect. 2.2.

### Capacitated facility location

For an overview on deterministic multi-period facility location and capacity expansion models, we also refer to the reviews by Melo et al. (2009) and Nickel and Saldanha-da Gama (2019).

Deterministic multi-period facility location and capacity expansion problems are often modelled with modular capacities. The expansion is then modelled as a jump between available capacity levels and leads to modification of existing facilities (Jena et al. 2015, 2016, 2017; Sauvey et al. 2020; Štádlerová and Schütz 2021; Štádlerová et al. 2022). Štádlerová and Schütz (2021) and Štádlerová et al. (2022) study a problem with modular capacities and piecewise linear short-term production costs (which can be seen as a combination of the problems studied by Correia and Captivo (2003) and Van den Broek et al. (2006)). Similar to Correia and Captivo (2003), they split investment and operational costs and provide specific operational costs to each modular capacity level. However, instead of one unit price for each capacity level, they model a capacity-specific piecewise linear short-term costs function similar to Van den Broek et al. (2006). Van den Broek et al. (2006) combine operational costs depending on installed capacity from Correia and Captivo (2003) with the linear staircase cost approximation from Holmberg (1994). Our modelling approach is identical to Štádlerová and Schütz (2021) and Štádlerová et al. (2022), as it enables us to model economies and dis-economies of scale in the investment and production processes having modular capacities.

Introducing demand uncertainty is a natural extension of deterministic problems. An early literature review on dynamic facility location and supply chain problems with stochastic parameters can be found in Owen and Daskin (1998). A review on facility location problems under uncertainty is provided by Snyder (2006) and recent summaries on facility location and supply chain problems under uncertainty are presented by Govindan et al. (2017), and Correia and Saldanha-da Gama (2019).

Traditionally, two-stage stochastic facility location problems are formulated as single-period problems. An early paper discussing a single-period capacitated facility location problem with random demand and non-linear cost function to model economies of scale is presented by Balachandran and Jain (1976). A generalization of their model is proposed by Schütz et al. (2008) who differentiate between general long-term costs for opening facilities and piecewise linear convex short-term costs for operating facilities. Correia and Melo (2021) study a two-stage multi-period facility location model with capacity expansion and reduction. Due to the complexity of the model, the problem can be solved for only 5 scenarios. The authors further show that using valid inequalities to strengthen the model improves computation times and optimality gaps.

Some supply chain design problems are characterized by a decision structure similar to two-stage facility location problems, as first-stage decisions are related to investments, while the second-stage decisions are related to demand allocation (Lucas et al. 2001). For a review on deterministic, as well as stochastic hydrogen supply chain design problems, we refer to Li et al. (2019). Kim et al. (2008) formulate the model of designing a hydrogen supply chain as a two-stage stochastic mixed-integer problem. Here, the first stage decision is related to investment in production facilities and storage while the second stage decision is related to demand allocation. The work by Almansoori and Shah (2012) and Nunes et al. (2015) can be seen as an extension of Kim et al. (2008) as the authors consider multiple time periods. Dayhim et al. (2014) present a two-stage stochastic problem for minimizing the total expected daily costs of the hydrogen supply chain facing uncertain demand. Unlike Kim et al. (2008) and Nunes et al. (2015), the authors consider also emission, risk and energy consumption costs. Similar to Nunes et al. (2015), Štádlerová et al. (2022) present a two-stage multi-period stochastic model to formulate the problem of locating hydrogen facilities. However, the authors extend the model by allowing capacity expansion in the second stage.

### Solution methods

Deterministic multi-period facility location and capacity expansion problems are in general hard to solve. The stochastic formulation might be closer to the real-world decision process, but also increases the complexity of the problem, especially when considering integer variables in the second stage. To find quality solutions for large instances, efficient solution algorithms need to be applied.

Lagrangian relaxation combined with heuristics for finding feasible solutions has performed well for deterministic multi-period facility location and capacity expansion problems (see, e.g., Shulman 1991; Jena et al. 2016, 2017; Štádlerová et al. 2022). Lagrangian relaxation has also been successfully used to solve single-period stochastic two-stage facility location problems with continuous second-stage variables (see, e.g., Schütz et al. 2008).

Sample average approximation (SAA) improves computational tractability by solving the problem repeatedly with a smaller number of scenarios (Kleywegt et al. 2001). Santoso et al. (2005) combine Benders decomposition with SAA to solve a supply chain design problem with uncertain demand and continuous second-stage variables. Sherali and Zhu (2006) and Angulo et al. (2016) study the application of Benders decomposition for stochastic problems with integer first and second-stage variables.

Nunes et al. (2015) and Štádlerová et al. (2022) apply SAA to solve the problem of locating hydrogen facilities. Nunes et al. (2015) solve the SAA problems with 15 scenarios. The number of scenarios in Štádlerová et al. (2022) is limited to 10 as the integer variables in the second stage make the problem harder to solve than the one studied in Nunes et al. (2015). SAA is often used in combination with other solution methods to further improve the quality of the solution (see, e.g., Santoso et al. 2005; Schütz et al. 2009; Li and Zhang 2018).

Researchers have only recently started to consider uncertainty in multi-period facility location problems with capacity expansion. Correia and Melo (2021) and Štádlerová et al. (2022) illustrate the challenges when using commercial software to solve two-stage stochastic programming models for this type of problem. To the best of our knowledge, our work is the first to present a solution method based on Lagrangian relaxation for the multi-period facility location problem with uncertain demand and capacity expansion in the second stage.

## Mathematical model

We formulate the problem of designing production infrastructure as a two-stage stochastic multi-period facility location and capacity expansion problem with modular capacities. In the first stage, we decide where and when to open new facilities along with their initial capacity levels. Once the demand is known in the second stage, we take decisions related to capacity expansion and demand allocation. The goal is to minimize the expected discounted total costs of satisfying the demand in each scenario.

### Problem definition

Candidate locations for production facilities are given by the set $\mathcal {I}$. The investment costs $C_{ik}$ for a new facility depend on location and installed capacity. The feasible production quantity at a facility depends on the installed capacity. The short-term production costs $F_{ibkt}$ then depend on location, installed capacity and its utilization, as well as time period. Customer locations are given by the set $\mathcal {J}$. For each customer, a specific demand, $D_{jt}^s$, is defined for each time period and each scenario *s* from the set $\mathcal {S}$. A customer may be served from one or more facilities. However, there are restrictions on which facility can serve which customers. For possible facility-customer combinations, unit distribution costs are based on distance. If the customers’ demand $D_{jt}^s$ cannot be satisfied, penalty costs $M^D$ apply for each unit of unsatisfied demand, denoted by $d_{jt}^s$. Penalty costs for unsatisfied demand can also be interpreted as additional costs for importing the product. If the quantities demanded from a facility are lower than the minimum production quantity for the installed capacity level, penalty costs $M^Q$ for a capacity excess unit $q_{it}^s$ apply. Similar to the penalty cost for unsatisfied demand, the capacity excess costs can be understood as costs for exporting excess production.

**Fig. 1:**
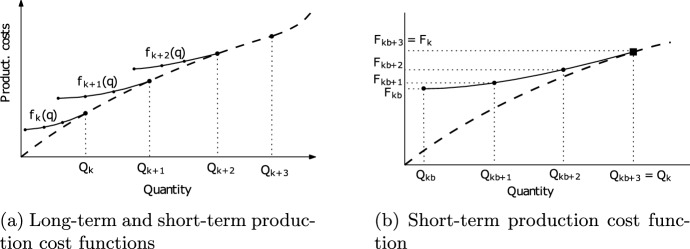
Long-term and short-term production cost functions

Once a facility has been opened, it cannot be closed. However, its capacity may be extended at a later time period to a higher capacity level. The expansion is allowed up to the highest available capacity ${\overline{K}}$. Capacity expansion leads to facility modification and it represents an expensive strategic decision. Thus, having a relatively short planning horizon, capacity expansion is allowed only once and then, the capacity cannot be changed until the end of the planning horizon ${\overline{T}}$. Investment costs $C_{ik}$ and expansion costs $E_{ikl}$ represent long-term costs and are separated from short-term production costs. For each capacity level, a specific convex piecewise linear short-term production cost function defines both the cost and the feasible production quantities for the installed capacity. Figure 1a exemplifies the link between long-term facility costs and short-term production costs. The short-term production cost function $f_k(q)$ for a specific capacity level *k* is illustrated in Fig. 1b, where $F_{kb}$ represents the production costs at a given breakpoint *b* of the piecewise linear cost function. The lowest breakpoint of the short-term production costs function represents the minimum production requirements for a given capacity level, while the highest breakpoint ${\overline{B}}_k$ corresponds to the installed capacity and thus to the upper production limit at capacity level *k*. The upper limit can only be increased by expansion towards a higher capacity level $k+n$. These capacity limits reflect the technological limitations of hydrogen production through electrolysis. This modelling approach is identical to Štádlerová et al. (2022), except for the addition of penalties for unsatisfied demand and excess production.

### Mathematical formulation

All used sets, parameters and decision variables are summarized below:

***Sets***

$$\mathcal {B}_k$$
Set of breakpoints of the short-term cost function connected to capacity level *k*, $\mathcal {B}_k = \{1,2,...,{\overline{B}}_k\}$;
$$\mathcal {I}$$
Set of candidate locations for production facilities;
$$\mathcal {J}$$
Set of customer locations;
$$\mathcal {K}$$
Set of available discrete capacity levels, $\mathcal {K} = \{1,2,...,{\overline{K}}\}$;
$$\mathcal {S}$$
Set of scenarios;
$$\mathcal {T}$$
Set of time periods, $\mathcal {T} = \{1,2,...,{\overline{T}}\};$
$$\mathcal {T}_1$$
Set of time periods corresponding to the first stage, $\mathcal {T}_1 \subset \mathcal {T}$.

***Parameters and coefficients***

$$C_{ik}$$
Investment costs at location $i \in \mathcal {I}$ for capacity level $k \in \mathcal {K}$;
$$D_{jt}^s$$
Demand at customer location $j \in \mathcal {J}$ in period $t \in \mathcal {T}$ and scenario $s \in \mathcal {S}$;
$$E_{ikl}$$
Costs of expanding at facility $i \in \mathcal {I}$ from capacity level $k \in \mathcal {K}$ to capacity level $l \in \mathcal {K}: l> k$;
$$F_{ibkt}$$
Production costs at facility $i \in \mathcal {I}$ at breakpoint $b \in \mathcal {B}_k$ at the short-term cost function of capacity level $k \in \mathcal {K}$ in period $t \in \mathcal {T}$;
$$L_{ij}$$
1 if demand at location $j \in \mathcal {J}$ can be served from facility $i \in \mathcal {I}$, 0 otherwise;
$$Q_{bk}$$
Production volume at breakpoint $b \in \mathcal {B}_k$ of the short-term cost function, for capacity level $k \in \mathcal {K}$;
$$T_{ij}$$
Distribution costs from facility $i \in \mathcal {I}$ to customer $j \in \mathcal {J}$;
$$M^D$$
Penalty costs for one unit of unsatisfied demand;
$$M^Q$$
Penalty costs for one excess unit;
$$y_{ikk0}$$
1 if a facility is opened at location $i \in \mathcal {I}$ with capacity level $k \in \mathcal {K}$ at the beginning of the planning horizon, 0 otherwise;
$$\delta _{t}$$
Discount factor in period $t \in \mathcal {T}$;
$$p^s$$
Probability of scenario $s \in \mathcal {S}$.

***Decision variables***

The mathematical model uses the following decision variables:

$$d_{jt}^s$$
Shortfall variable: amount of not satisfied demand at customer location $j \in \mathcal {J}$ in period $t \in \mathcal {T}$ for scenario $s \in \mathcal {S}$;
$$q_{it}^s$$
Capacity excess variable: amount of production excess units from facility location $i \in \mathcal {I}$ in period $t \in \mathcal {T}$ for scenario $s \in \mathcal {S}$ that is not distributed to customers;
$$x_{ijkt}^s$$
Amount of customer demand at location $j \in \mathcal {J}$ satisfied from facility $i \in \mathcal {I}$ operating at capacity level $k \in \mathcal {K}$ in period $t \in \mathcal {T}$ in scenarios $s \in \mathcal {S}$;
$$y_{iklt}^s$$
1 if facility is operated at location $i \in \mathcal {I}$, originally opened at capacity level $k \in \mathcal {K}$, and operating at capacity level $l\in \mathcal {K}: l \ge k$ in period $t \in \mathcal {T}$ and scenario $s \in \mathcal {S}$, 0 otherwise;
$$\mu _{bilt}^s$$
Weight of breakpoint $b \in \mathcal {B}_l$ at location $i \in \mathcal {I}$ for capacity level $l \in \mathcal {K}$ in period $t \in \mathcal {T}$ and scenario $s \in \mathcal {S}$.

We present a two-stage stochastic multi-period Mixed-Integer-Programming (MIP) model. The model is similar to Štádlerová et al. (2022), but additionally provides relatively complete recourse, as we introduce variables for unsatisfied demand and capacity excess. The model is given as:

1 $$\begin{aligned} \begin{aligned}&\min \sum _{s \in \mathcal {S}} p^s \left[ \sum _{i \in \mathcal {I}} \sum _{k \in \mathcal {K}}\sum _{t \in \mathcal {T}}\delta _{t} C_{ik}\left( y_{ikkt}^s-y_{ikk(t-1)}^s\right) \right. \\&\quad +\left. \sum _{i \in \mathcal {I}}\sum _{k \in \mathcal {K}}\sum _{l \in \mathcal {K}: l > k} \sum _{t \in \mathcal {T}}\delta _{t} E_{ikl} \left( y_{iklt}^s - y_{ikl(t-1)}^s \right) \right. \\&\quad +\left. \sum _{b \in \mathcal {B}} \sum _{i \in \mathcal {I}}\sum _{l \in \mathcal {K}} \sum _{t \in \mathcal {T}}\delta _{t}F_{iblt} \mu _{bilt}^s + \sum _{i \in \mathcal {I}} \sum _{j \in \mathcal {J}}\sum _{l \in \mathcal {K}}\sum _{t \in \mathcal {T}}\delta _{t}T_{ij} x_{ijlt}^s \right. \\&\quad \left. \sum _{j \in \mathcal {J}}\sum _{t \in \mathcal {T}} M^D d_{jt}^s + \sum _{i \in \mathcal {I}}\sum _{t \in \mathcal {T}}M^Q q_{it}^s \right] \end{aligned} \end{aligned}$$

subject to:

2 $$\begin{aligned} \quad \sum _{k \in \mathcal {K}} \sum _{l \in \mathcal {K}: l \ge k}y_{iklt}^s \le 1,{} & {} i \in \mathcal {I}, t \in \mathcal {T}, s \in \mathcal {S}, \end{aligned}$$

3 $$\begin{aligned} \quad \sum _{k \in \mathcal {K}} \sum _{l \in \mathcal {K}: l > k}y_{iklt}^s = 0,{} & {} i \in \mathcal {I}, t \in \mathcal {T}_1, s \in \mathcal {S}, \end{aligned}$$

4 $$\begin{aligned} \quad \sum _{t^{\prime } = 1}^{t-1}y_{ikkt^{\prime }}^s \ge \sum _{l \in \mathcal {K}: l > k}y_{iklt}^s,{} & {} i \in \mathcal {I}, k \in \mathcal {K}, t \in \mathcal {T}, s \in \mathcal {S}, \end{aligned}$$

5 $$\begin{aligned} \quad \sum _{l \in \mathcal {K}: l \ge k} y_{iklt}^s \ge \sum _{l \in \mathcal {K}: l \ge k} y_{ikl(t-1)}^s,{} & {} i \in \mathcal {I}, k \in \mathcal {K}, t \in \mathcal {T}, s \in \mathcal {S} \end{aligned}$$

6 $$\begin{aligned} \quad y_{iklt}^s- y_{ikl(t-1)}^s \ge 0,{} & {} i \in \mathcal {I}, k \in \mathcal {K}, l \in \mathcal {K}:l > k, t \in \mathcal {T}, s \in \mathcal {S}, \end{aligned}$$

7 $$\begin{aligned} \quad \sum _{b \in \mathcal {B}_l}\mu _{bilt}^s = \sum _{k \in \mathcal {K}}y_{iklt}^s,{} & {} i \in \mathcal {I}, l \in \mathcal {K}, t \in \mathcal {T}, s \in \mathcal {S}, \end{aligned}$$

8 $$\begin{aligned} \quad \sum _{j \in \mathcal {J}}\sum _{l \in \mathcal {K}} x_{ijlt}^s + q_{it}^s = \sum _{b \in \mathcal {B}_l}\sum _{l \in \mathcal {K}}Q_{bl}\mu _{bilt}^s,{} & {} i \in \mathcal {I}, t \in \mathcal {T}, s \in \mathcal {S}, \end{aligned}$$

9 $$\begin{aligned} \quad \sum _{i \in \mathcal {I}}\sum _{l \in \mathcal {K}}x_{ijlt}^s + d_{jt}^s = D_{jt}^s,{} & {} j \in \mathcal {J}, t \in \mathcal {T}, s \in \mathcal {S}, \end{aligned}$$

10 $$\begin{aligned} \quad x_{ijlt}^s \le L_{ij} D_{jt}^s \sum _{k \in \mathcal {K}: k \le l}y_{iklt}^s,{} & {} i \in \mathcal {I}, j \in \mathcal {J}, l \in \mathcal {K}, t \in \mathcal {T}, s \in \mathcal {S}, \end{aligned}$$

11 $$\begin{aligned} \quad \frac{1}{|\mathcal {S}|}\sum _{s^{\prime } \in \mathcal {S}}\sum _{l \in \mathcal {K}: l \ge k}y_{iklt}^{s^{\prime }}= \sum _{l \in \mathcal {K}: l \ge k}y_{iklt}^s,{} & {} i \in \mathcal {I}, k \in \mathcal {K}, t \in \mathcal {T}, s \in \mathcal {S}, \end{aligned}$$

12 $$\begin{aligned} \quad y_{iklt}^s \in \{0,1\},{} & {} i \in \mathcal {I}, k \in \mathcal {K}, l \in \mathcal {K}: l \ge k, t \in \mathcal {T}, s \in \mathcal {S}, \end{aligned}$$

13 $$\begin{aligned} \quad x_{ijlt}^s \ge 0,{} & {} i \in \mathcal {I}, j \in \mathcal {J},l \in \mathcal {K}, t \in \mathcal {T}, s \in \mathcal {S} \end{aligned}$$

14 $$\begin{aligned} \quad \mu _{bilt}^s \ge 0,{} & {} b \in \mathcal {B}_l, i \in \mathcal {I}, k \in \mathcal {K}, t \in \mathcal {T}, s \in \mathcal {S}, \end{aligned}$$

15 $$\begin{aligned} \quad q_{it}^s \ge 0,{} & {} i \in \mathcal {J}, t \in \mathcal {T}, s \in \mathcal {S}, \end{aligned}$$

16 $$\begin{aligned} \quad d_{jt}^s \ge 0,{} & {} j \in \mathcal {J}, t \in \mathcal {T}, s \in \mathcal {S}. \end{aligned}$$

Objective (1) minimizes the expected discounted sum of investment, expansion, production, and distribution costs as well as the penalty costs for unsatisfied demand and excess capacity. Constraints (2) state that for each time period and scenario, at most one facility can be operated at a given location. Constraints (3) ensure that in the first stage, facilities can be only opened, but not expanded, while Inequalities (4) only allow expansion of opened facilities. Constraints (5) ensure that once a facility is opened, it cannot be closed, but only expanded, while Constraints (6) require that an open facility can only be expanded once during the planning horizon. Equalities (7) link capacity level *k* with the appropriate short-term cost function and ensure that only opened facilities can be used for production. Constraints (8) ensure that the whole production is either distributed to customers or allocated to the capacity excess variable. The constraints also implicitly assure the minimum production requirements through the quantity $Q_{bl}$ given by the smallest breakpoint *b*. Note that this formulation is also applicable for problems without minimum production requirements, as we can define the quantity belonging to the smallest breakpoint as zero. Equations (9) ensure that demand is satisfied or registered as demand shortfall. Restrictions (10) are formulated in the form of strong inequalities. They limit which facility can satisfy which customer and link the distribution variable to the operated capacity level. Such linking constraints provide stronger bounds and lead to lower integrality gaps from linear relaxation than aggregated linking constraints (see, e.g., Jena et al. 2016). Constraints (11) are the non-anticipativity constraints that ensure that the opening capacity level *k* is the same for all scenarios while the operating capacity level *l* is scenario specific. Constraints (12)–(16) are the non-negativity and binary requirements.

## Lagrangian relaxation

In the domain of facility location, Lagrangian relaxation has mostly been applied in deterministic settings (see, e.g., Shulman 1991; Jena et al. 2016, 2017; Štádlerová et al. 2022). Given the similar structure of the here considered facility location problem, Lagrangian relaxation remains an attractive candidate, even when considering multiple demand scenarios. We now present the Lagrangian heuristic used to solve our stochastic problem. Specifically, we relax demand constraints (9) which are the only constraints connecting the decision variables among the different facility locations and have been a popular choice in the literature (Shulman 1991; Schütz et al. 2008; Jena et al. 2016). We define $\lambda _{jt}^s$ as the matrix of Lagrangian multipliers and we obtain the following Lagrangian subproblem:

17 $$\begin{aligned} \begin{aligned} LR(\varvec{\lambda })&= \min \sum _{s \in \mathcal {S}} p^s \left[ \sum _{i \in \mathcal {I}}\sum _{k \in \mathcal {K}}\sum _{t \in \mathcal {T}}\delta _{t} C_{ik}\left( y_{ikkt}^s-y_{ikk(t-1)}^s\right) \right. \\&\quad + \left. \sum _{i \in \mathcal {I}} \sum _{k \in \mathcal {K}}\sum _{l \in \mathcal {K}:l > k}\sum _{t \in \mathcal {T}}\delta _{t} E_{ikl} (y_{iklt}^s - y_{ikl(t-1)}^s) \right. \\&\quad +\left. \sum _{i \in \mathcal {I}} \sum _{b \in \mathcal {B}_l}\sum _{l \in \mathcal {K}}\sum _{t \in \mathcal {T}}\delta _{t}F_{iblt} \mu _{bilt}^s + \sum _{i \in \mathcal {I}} \sum _{t \in \mathcal {T}}M^Q q_{it}^s \right. \\&\quad +\left. \sum _{i \in \mathcal {I}} \sum _{j \in \mathcal {J}}\sum _{l \in \mathcal {K}}\sum _{t \in \mathcal {T}}(\delta _{t}T_{ijt} - \lambda _{jt}^s)x_{ijlt}^s \right. \\&\quad \left. +\sum _{j \in \mathcal {J}}\sum _{t \in \mathcal {T}} (M^D - \lambda _{jt}^s) d_{jt}^s + \sum _{j \in \mathcal {J}}\sum _{t \in \mathcal {T}} \lambda _{jt}^s D_{jt}^s \right] , \end{aligned} \end{aligned}$$

subject to Constraints (2)–(8) and (10)–(15). In the relaxed problem, the variable $d_{jt}$ is unbounded and it has no connection to any other decision variable. Since we have a minimization problem, it can be shown that the term $\sum _{j \in \mathcal {J}}\sum _{t \in \mathcal {T}} (M^D - \lambda _{jt}^s) d_{jt}^s$ becomes zero in any optimal solution and can hence be omitted. Further, for given multipliers $\lambda _{jt}^s$, the expression $\sum _{s \in \mathcal {S}}\sum _{j \in \mathcal {J}}\sum _{t \in \mathcal {T}}p^s \lambda _{jt} D_{jt}^s$ is constant. As all constraints are defined separately for each facility location $i \in \mathcal {I}$, we can decompose the problem and solve it independently for each facility location $i \in \mathcal {I}$. We can then define $LR(\lambda )=\sum _{i\in \mathcal {I}}g_i(\lambda ) + \sum _{s \in \mathcal {S}}\sum _{j \in \mathcal {J}}\sum _{t \in \mathcal {T}}p^s \lambda _{jt} D_{jt}^s$ where $g_i(\lambda )$ is the optimal value of the Lagrangian subproblem for location *i*:

18 $$\begin{aligned} \begin{aligned} \quad g_{i}(\lambda )&= \min \sum _{s \in \mathcal {S}} p^s \left[ \sum _{k \in \mathcal {K}}\sum _{t \in \mathcal {T}}\delta _{t} C_{ik}\left( y_{ikkt}^s-y_{ikk(t-1)}^s\right) \right. \\&\quad \left. +\sum _{k \in \mathcal {K}}\sum _{l \in \mathcal {K}:l > k}\sum _{t \in \mathcal {T}}\delta _{t} E_{ikl} (y_{iklt}^s - y_{ikl(t-1)}^s ) \right. \\&\quad \left. +\sum _{b \in \mathcal {B}_l} \sum _{l \in \mathcal {K}}\sum _{t \in \mathcal {T}}\delta _{t}F_{iblt} \mu _{bilt}^s + \sum _{t \in \mathcal {T}}M^Q q_{it}^s x \right. \\&\quad \left. +\sum _{j \in \mathcal {J}}\sum _{l \in \mathcal {K}}\sum _{t \in \mathcal {T}}(\delta _{t}T_{ijt} - \lambda _{jt})x_{ijlt}^s \right] , \end{aligned} \end{aligned}$$

subject to constraints (2)–(8) and (10)–(15) defined for the specific facility $i \in \mathcal {I}$.

### Solving the Lagrangian subproblem

The optimal solution to the Lagrangian subproblem represents the optimal opening and expansion schedule and capacity level for each facility all scenarios and over all scenarios such that the expected total costs (18) are minimized. In deterministic settings, such schedules have been found by solving a shortest path problem via dynamic programming (see, e.g., Shulman 1991; Jena et al. 2016; Štádlerová et al. 2022). Given that, in our two-stage stochastic problem, the expansion schedule (i.e, the second-stage decisions) may be different for each scenario, shortest path networks including all opening and expansion decisions would be too complex and computationally intractable. Our approach, therefore, evaluates the optimal expansion schedule for all possible opening decisions separately. Specifically, for each opening capacity level and time period (i.e, the first-stage decisions), the shortest path problem is solved via dynamic programming independently for each scenario starting from the defined opening time period and capacity level, similar to Shulman (1991), Jena et al. (2016) and Štádlerová et al. (2022). For each scenario, at most one capacity expansion is allowed. The shortest path problem for solving the Lagrangian subproblem is detailed in Sect. 4.1.2. For a given capacity level, time period, and scenario, the problem of customer allocation then becomes a continuous knapsack problem which is explained next.

#### Continuous knapsack

The costs of the optimal demand allocation for a given capacity level $l \in \mathcal {K}$, time period $t \in \mathcal {T}$ and scenario $s \in \mathcal {S}$ can be computed by solving a continuous knapsack problem with piecewise linear costs (Amiri 1997; Christensen and Klose 2021). The costs of the continuous knapsack for a given capacity level *l* consist of production costs, penalty costs for capacity excess and reduced distribution costs. The costs are calculated for a given facility $i \in \mathcal {I}$, capacity level *l*, time period *t* and scenario *s*. Since the continuous knapsack is calculated for a given capacity level *l*, the strong inequalities (10) are considered in the calculation of the knapsack costs. We formulate the continuous knapsack problem as:

19 $$\begin{aligned} K_{ilt}^s(\varvec{\lambda }) = \min \sum _{b \in \mathcal {B}_l}F_{iblt}\mu _{bilt}^s + M^Q q_{it}^s + \sum _{j \in \mathcal {J}}(T_{ij} - \lambda _{jt}^s)x_{ijlt}^s, \end{aligned}$$

subject to:

20 $$\begin{aligned} \quad x_{ijlt}^s \le L_{ij}D_{jt}^s,{} & {} j \in \mathcal {J}, \end{aligned}$$

21 $$\begin{aligned} \quad \sum _{j \in \mathcal {J}}x_{ijlt}^s + q_{it}^s = \sum _{b \in \mathcal {B}_l}Q_{bl}\mu _{bilt}^s,{} & {} \end{aligned}$$

22 $$\begin{aligned} \quad \sum _{b \in \mathcal {B}_l}\mu _{bilt}^s = 1,{} & {} \end{aligned}$$

23 $$\begin{aligned} \quad q_{it}^s \ge 0,{} & {} \end{aligned}$$

24 $$\begin{aligned} \quad x_{ijlt}^s \ge 0,{} & {} j \in \mathcal {J}, \end{aligned}$$

25 $$\begin{aligned} \quad \mu _{bilt}^s \ge 0,{} & {} b \in \mathcal {B}_l. \end{aligned}$$

The problem (19) – (25) is similar to the one solved by Schütz et al. (2008). However, in contrast to Schütz et al. (2008), we have a minimum production requirement for each capacity level and allow for capacity excess. For a given capacity level $l \in \mathcal {K}$, period $t \in \mathcal {T}$ and scenario $s \in \mathcal {S}$, we calculate the unit production costs as $u_{ilbt} = \frac{F_{ib+1lt}-F_{iblt}}{Q_{b+1\,l}-Q_{bl}}$. We further define the marginal costs of serving one additional demand unit as: $m_{ijlbt}^s = T_{ij} - \lambda _{ij}^s$+ $u_{ilbt}$. Note that the marginal costs are dependent on the line-piece of the short-term cost function. For each customer, we calculate the reduced costs $T_{ij}-\lambda _{ij}^s$ and start allocating customers with the lowest reduced costs until $m_{ijlbt}^s > 0$ for the first time or until the capacity limit of the line-piece $Q_{b+1l}$ is reached. For the next line-piece, the marginal costs must be updated. However, the ordering of customers according to their reduced costs remains unchanged. We continue adding customers until $m_{ijlbt}^s > 0$ or until the capacity limit $Q_{{\overline{B}}_l}$ is reached.

If the minimum production requirement for a given capacity cannot be fulfilled with customers with negative reduced costs, we may also have to add customers with positive reduced costs. Assuming that penalty costs are always higher than the costs of satisfying customers with positive reduced costs, we prefer customers with positive reduced costs to using variables $q_{it}^s$. However, if there are no more customers that could be added and the minimum production requirement is still not satisfied, we can use variables $q_{it}^s$ that allow satisfying the minimum production requirement for penalty costs. If the penalty costs are sufficiently high, a capacity decision leading to $q_{it}^s > 0$ will most likely not be optimal since demand does not need to be satisfied in the relaxed problem.

#### Formulating the shortest path problem

As previously mentioned, in deterministic problems, the problem of finding the optimal opening and expansion decision can be formulated as a shortest path problem in a single graph and solved via dynamic programming (Shulman 1991; Jena et al. 2016; Štádlerová et al. 2022). In our scenario-based stochastic problem, such a single graph formulation is not suitable, since the opening decision has to remain the same for all scenarios, but the expansion decision can be different for each scenario. Therefore, we define one shortest path problem for each tuple $({\overline{k}}_0, {\overline{t}}_0)$ of opening capacity level ${\overline{k}}_0 \in \mathcal {K} \cup \{0\}$ and opening time period ${\overline{t}}_0 \in \mathcal {T}$. For each given $({\overline{k}}_0,{\overline{t}}_0)$, the second stage problem is then separable in scenarios and we can calculate the shortest path problem separately for each scenario. Finally, we choose the first-stage opening decision that leads to the lowest expected costs over the shortest path problems.

For given opening decision $({\overline{k}}_0,{\overline{t}}_0)$, let $C^E({\overline{k}}_0, {\overline{t}}_0)$ denote the costs of the expected shortest path. The costs of opening and operating a facility during the opening period are equal to the investment costs and the expected costs of the continuous knapsack: $\delta _{t_0} C_{{\overline{k}}_0{\overline{t}}_0} + \sum _{s \in \mathcal {S}} \delta _{t_0}p^{s}K_{i{\overline{k}}_0{\overline{t}}_0}^{s}(\varvec{\lambda })$. The total costs can then be written as: $\delta _{t_0} C_{i{\overline{k}}_0{\overline{t}}_0} + \sum _{s \in \mathcal {S}} \delta _{t_0}p^{s}K_{i{\overline{k}}_0{\overline{t}}_0}^{s}(\varvec{\lambda }) + C^E({\overline{k}}_0 {\overline{t}}_0)$.

**Fig. 2:**
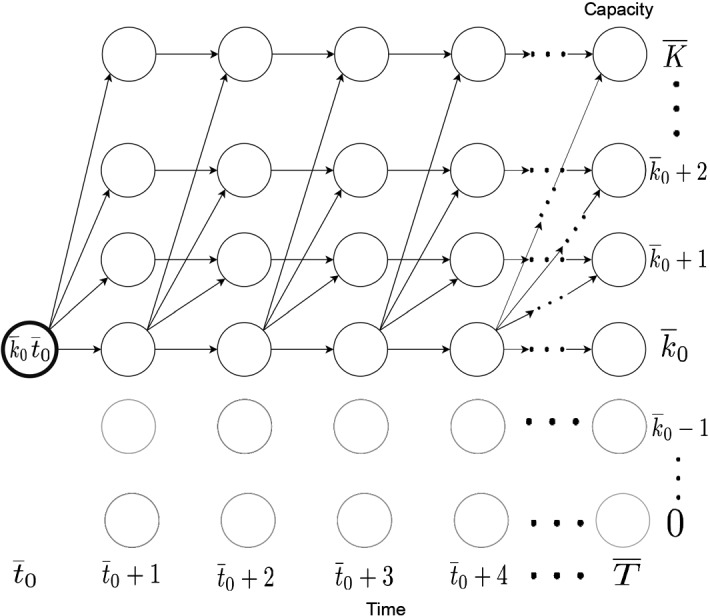
Structure of the shortest path problem for a given investment decision and scenario

For given opening decision $({\overline{k}}_0,{\overline{t}}_0)$ and given scenario $s \in \mathcal {S}$, the graph structure is illustrated in Fig. 2. Let $l_{{\overline{T}}}$ denote the capacity level at the end of the planning horizon. The graph shows that after investing in capacity level ${\overline{k}}_0$, we can either keep the capacity at level ${\overline{k}}_0$ or we can expand once during the planning horizon towards a higher capacity level $l_{{\overline{T}}} \in \mathcal {K}: l_{{\overline{T}}} > {\overline{k}}_0$. Note that all capacities larger than ${\overline{k}}_0$ are available for expansion. However, we are not allowed to reduce the capacity level below the level given by ${\overline{k}}_0$.

The costs for an arc from node $(k,t-1)$ to node $(k^\prime ,t)$ in our graph are given as:

$$\begin{aligned} C(k,t-1)(k^{\prime },t) =\left\{ \begin{array}{lll} E_{ikk^{\prime }} + K_{ik^{\prime }t}(\lambda ) &{} \text {if} \quad k = {\overline{k}}_{0} \wedge k^{\prime } = l_{{\overline{T}}},&{} \qquad (26) \\ K_{ik^{\prime }t}(\lambda ) &{} \text {if} \quad k = k^{\prime },&{} \qquad (27) \\ +\infty &{} \text {else}.&{}\qquad (28) \end{array}\right. \end{aligned}$$

Equation (26) calculates the costs of expanding a facility as the sum of expansion costs $E_{ikk^{\prime }}$ and the costs of continuous knapsack $K_{ik^{\prime }t}(\varvec{\lambda })$. Equation (27) calculates the costs of operating the facility if there is no change in the installed capacity level. The short-term production costs are then given as the costs of the continuous knapsack $K_{ik^{\prime }t}(\varvec{\lambda })$. We define the costs of all other combinations as $+\infty$ (28) as these are infeasible and hence can be omitted in the graph structure.

### Updating the Lagrangian multipliers

The lower bound on the Objective (1) is given by solving (??) subject to Constraints (2)–(8) and (10)–(15) for given multipliers $\lambda _{jt}^s$. In order to find the highest possible lower bound, we have to find a $\varvec{\lambda }$ that maximizes the Lagrangian dual problem: $LD = \max _{\lambda }LR(\varvec{\lambda })$. To solve the LD problem, we iteratively use the box step method (Marsten et al. 1975) similar to Schütz et al. (2009) and Štádlerová et al. (2022), as this method allows us to update the multipliers without computing an upper bound. We calculate the subgradient $\nabla _{jt}^{ms}$ as $\nabla _{jt}^{ms} = D_{jt}^s - \sum _{i \in \mathcal {I}}x_{ijt}^s$ in each iteration *m* and for each scenario *s*. We then define $L^m = LR(\varvec{\lambda }^m)-\sum _{j \in \mathcal {J}} \sum _{t \in \mathcal {T}}\sum _{s \in \mathcal {S}} p^s \lambda _{jt}^{ms} \nabla _{jt}^{ms}$ and find the updated multipliers by solving the following linear optimization problem:

29 $$\begin{aligned} \quad \max \phi \end{aligned}$$

30 $$\begin{aligned} \quad \phi \le L^i + \sum _{j \in \mathcal {J}} \sum _{t \in \mathcal {T}} \sum _{s \in \mathcal {S}} p^s \nabla _{jt}^{is} \lambda _{jt}^{m+1,s}{} & {} i = 1, ..., m, \end{aligned}$$

31 $$\begin{aligned} \quad \lambda _{jt}^{m+1,s} \le \lambda _{jt}^{ms} + \Delta _{jt}^{ms}{} & {} j \in \mathcal {J}, t \in \mathcal {T}, s \in \mathcal {S}, \end{aligned}$$

32 $$\begin{aligned} \quad \lambda _{jt}^{m+1,s} \ge \lambda _{jt}^{ms} - \Delta _{jt}^{ms}{} & {} j \in \mathcal {J}, t \in \mathcal {T}, s \in \mathcal {S}, \end{aligned}$$

33 $$\begin{aligned} \quad \phi \in \mathbb {R}, \lambda _{jt}^{m+1,s} \in \mathbb {R}. \end{aligned}$$

We limit how much the Lagrangian multipliers can change in each iteration using box constraints (31) and (32). These boxes are specific for each variable $\lambda _{jt}^s$. If the sign of the subgradient $\nabla _{jt}^{ms}$ changes from the previous iteration $m-1$, we decrease the box size as: $\Delta _{jt}^{ms} = \alpha \Delta _{jt}^{ms}$, where $0< \alpha < 1$ (Štádlerová et al. 2022). The aim of reducing the box size is to speed up the procedure of finding the optimal multipliers (Marsten et al. 1975). If the multipliers do not change for three consecutive iterations, we reset the box size and allow large changes of the multipliers again in order to escape a local optimum.

### Upper bound

**Fig. 3:**
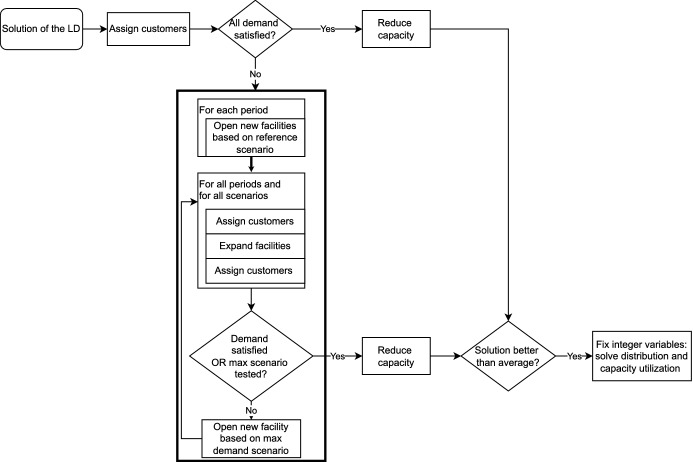
Upper bound structure

We use a greedy heuristic to build a feasible solution based on the solution of the relaxed problem (i.e., the LD). Due to capacity excess and shortfall variables, the solution to the relaxed problem is always feasible. However, these variables may imply high penalty costs. In our upper bound heuristic, we aim to find first-stage solutions that are feasible in all scenarios without or with minimal penalty costs. The heuristic is an extension of the deterministic solution method presented by Štádlerová et al. (2022). The main steps of the heuristic are illustrated in Fig. 3.

We initialize the solution using the installed capacity from the *Solution of the LD*, i.e., the capacity level of opened facilities. The allocation and distribution decisions from the relaxed problem are ignored when we assign customers to facilities. Note that the solution of the relaxed problem satisfies the non-anticipativity constraints, so customers can be assigned to facilities separately for each scenario.

In step *Assign customers*, for a given scenario and time period, we create pairs of available facilities $i \in \mathcal {I}$ and unsatisfied customers $j \in \mathcal {J}$. These pairs are sorted in increasing order of their reduced transportation costs $T_{ij} - \lambda _{jt}^s$. We start with the pair with the lowest reduced transportation costs and serve the unsatisfied customer from the corresponding facility. We repeat this step until all available capacity is used or the demand of all customers is satisfied.

Within step *Assign customers*, we also verify the minimum production requirements and try to fix them. If the minimum production requirements of facility B are not satisfied, the heuristic selects a facility with high utilization A. If there are customers that can be satisfied both from A and B, the heuristic uses facility A to shift some of its production to facility B until the production is sufficient. Otherwise, we have to find facility C, which has common customers with both A and B. Then, we shift some production from facility A via auxiliary facility C to facility B. The heuristic uses up to three auxiliary facilities to shift production between A and B. When shifting the production quantities from A to B, customers are sorted in increasing order based on their reduced transportation costs from facility B. We start reallocating customers with the lowest reduced transportation costs. After reallocation, the spare capacity in facility A is used to satisfy additional unsatisfied customers.

The capacity obtained from the solution of the relaxed problem is most likely not sufficient to satisfy demand in all scenarios. If there are unsatisfied customers after step *Assign customers* considering *Solution of the LD*, the upper bound heuristic increases the capacity to satisfy all customers or to minimize the penalty costs for demand shortfall. These steps are illustrated in Fig. 3 in the bold frame.

In general, a new facility can be opened at a location without a facility. Expansion is allowed only at a location with an existing facility that has not been expanded yet. When selecting the facility that has to be opened or expanded, there are usually several candidates. We choose the candidate that can satisfy most of the unsatisfied customers. In case of a tie, we prioritize the facility with lower production costs. The chosen capacity for opening or expanding a facility is the lowest possible capacity level that can satisfy the demand.

We execute the upper bound heuristic repeatedly for 4 different reference scenarios: maximum, minimum, mean and median demand scenarios and then, we select the solution with the lowest objective. Since the opening decision has to be equal for all scenarios, we start with step *Open new facilities based on reference scenario* and implement the first-stage decisions based on the chosen reference scenario for all other scenarios before executing routines that are specific for each scenario. Considering only 4 reference scenarios enables shorter computation times compared to evaluating first-stage decisions of each scenario. Simultaneously, first-stage decisions provided by one of the reference scenarios have shown to be sufficiently good for our upper bound.

After the opening decisions are fixed, step *Assign customers* can be again performed for each scenario independently as well as the expansion decisions in the step *Expand facilities* since these are the second-stage decisions. If the installed capacity is still not sufficient and our reference scenario differs from the maximum demand scenario, the heuristics performs the step *Open new facility based on max demand scenario*, where the opening decisions are taken based on unsatisfied customers in the scenario with maximum demand. Then, the capacity installed in the first stage increases in all scenarios. Note that these new facilities can later be expanded as well.

The upper bound heuristic aims to install sufficient capacity to avoid penalties for demand shortfall. However, the solution of the LD as well as in the upper bound heuristic may have installed more capacity than necessary. Therefore, we try to reduce the installed capacity or remove some facilities in order to improve the total costs. We first try to remove facilities with capacity excess. Specifically, we identify a facility that causes penalties for capacity excess and check whether the allocated customers can be served from other opened facilities in all time periods and scenarios. These facilities need to have some spare capacity and satisfy the distance limit to the customers. If all customers can be reallocated, we remove the facility. Further, we extend the deterministic procedure from Jena et al. (2016) to our stochastic problem. We fix the demand allocation decisions, and use a dynamic programming algorithm, to find optimal opening and expansion capacities and time periods to satisfy the given quantities.

If the obtained solution is better than the average of the previously found solutions, we fix all integer variables and solve a problem consisting of demand allocation and facility utilization with Gurobi. When evaluating the average costs, we consider objectives before re-optimizing distribution and facility utilization. This enables us to save time, as we do not need to re-optimize all available solutions and reduce the risk of ignoring a potentially good solution.

## Case study

In this section, we introduce the input data for our case study from Norway. The case reflects the real-world problem of producing hydrogen for the Norwegian transport sector. However, our model is applicable to variety of facility location and capacity expansion planning problems.

### Candidate locations and production costs

We consider 17 ports along the Norwegian coast as candidate locations derived from the interactive map provided by Ocean Hyway Cluster (2020b). For testing purposes, we further extend the number of candidate locations to 34. All these locations are Norwegian ports and contain the original 17 locations as a subset.

In our case study, we consider alkaline electrolysis as production technology since it is as of today the most mature and the cheapest available technology. For alkaline electrolysers, minimum production requirements must be considered (Andrenacci et al. 2022). We assume that investment costs are the same for all facility locations. We approximate the long-term production cost function with 8 and 16 modular capacity levels, each with specific investment costs. For 16 capacity levels, each of the original 8 capacity levels is split into two levels. The investment costs for 8 capacity levels are shown in Table 1. All investment costs are calculated based on the model by Jakobsen and Åtland (2016).

**Table 1:** Investment costs for electrolysis (Štádlerová and Schütz 2021)

Discrete capacity	1	2	3	4	5	6	7	8
Capacity (tonnes/day)	0.6	3.1	6.2	12.2	30.3	61.0	151.5	304.9
Investment (mill. €)	1.4	6.0	11.2	20.5	46.5	87.2	197.7	371.5

Electrolysis production costs are highly dependent on electricity prices. Even though Norway is split into 5 electricity price regions, the prices differ mainly between the northern part and the southern part of Norway. We, therefore, use different production costs dependent on whether the candidate locations are in northern Norway (N) or southern Norway (S). All candidate locations situated in and north of Trondheim are considered to belong to the northern region.

To calculate the production costs in periods 1 to 9, we use the 2021 yearly average electricity prices for the two regions. On average, the prices in the southern region were 1.8 times higher than prices in the northern region in 2021 (Nord Pool AS 2022). For periods 10 to 14, we use the electricity price based on the forecast from NVE (2021) that predicts a smaller difference between the northern and the southern region. According to this forecast, the price in the southern region should be about 1.2 times higher than in the northern region. The production costs are calculated using the model by Jakobsen and Åtland (2016). The production costs at $100\%$ capacity utilization for southern (S) and northern Norway (N) are shown in Table 2.

**Table 2:** Production costs for EL at $100\%$ capacity utilization

Discrete capacity	1	2	3	4	5	6	7	8
Capacity (tonnes/day)	0.6	3.1	6.2	12.2	30.3	61.0	151.5	304.9
Production S (€/kg)	4.26	4.21	4.20	4.18	4.16	4.14	4.13	4.11
Production N (€/kg)	2.54	2.50	2.47	2.46	2.44	2.42	2.40	2.39

For each capacity level, we approximate the short-term production cost function by a piecewise linear function with 4 breakpoints. The production range for electrolysis is $15\% - 100\%$ (NEL Hydrogen 2018). Thus, we define breakpoints of the short-term cost function at $15 \%$, $50 \%$, $80 \%$, and $100 \%$ of the installed capacity level. For each capacity level, the $15 \%$ breakpoint represents the minimum production requirement.

### Penalty costs

We define penalty costs for each unit of demand shortfall and capacity excess. Since the focus of this case study is on domestic hydrogen production for domestic customers, we set high penalties for both demand shortfall and capacity excess of $10^9$ €/kg to avoid both import and export of hydrogen.

### Distribution costs

Hydrogen is distributed in trucks as pipelines are not a suitable distribution solution for Norway. Distribution costs per kilometer and kilogram of hydrogen are based on the hydrogen distribution study provided by Danebergs and Aarskog (2020) and taken from Štádlerová and Schütz (2021). The distribution costs are defined for different distance intervals as shown in Table 3. The maximum distance between production facilities and customers is 1000 km.

**Table 3:** Hydrogen distribution costs in [€/km/kg H$_2$] (Štádlerová and Schütz 2021)

Distance (km)	1–50	51–100	101–200	201–400	401–800	801–1000
Costs	0.00498	0.00426	0.00390	0.00372	0.00363	0.00360

### Demand

The total hydrogen demand consists of three components:

- Maritime demand (Ocean Hyway Cluster 2020a),
- Land-based demand (DNV GL 2019),
- Offshore demand (Aglen and Hofstad 2022).

The maritime demand is based on public contracts for high-speed passenger ferries and car ferries. This component is considered to be deterministic and is present in all demand scenarios. In the land-based and offshore sectors, the future demand share among competing zero-emission carriers is highly uncertain. Thus, the demand share, and as such the total demand for hydrogen, from these sectors differs in each scenario. The deterministic demand estimations in DNV GL (2019) and Aglen and Hofstad (2022) are highly uncertain and we consider them to represent the maximal potential demand for hydrogen in these sectors.

**Fig. 4:**
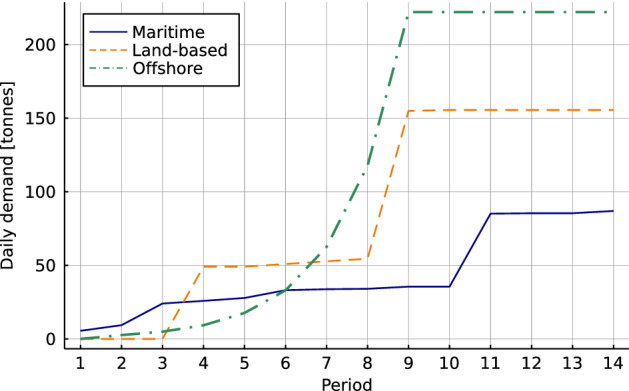
Annual daily demand

Figure 4 shows the evolution of the maximum potential demand for all demand components over the planning horizon. Maritime demand is characterized by a steady demand increase, and the demand jump in time period 11 represents ships on the coastal route Bergen-Kirkenes that will switch to hydrogen fuel as well. The transition towards hydrogen in the land-based sector is expected to come in two waves that cause the demand jumps in periods 4 and 9. In the offshore sector, most of the ships should be transformed to use hydrogen fuel before period 10. Afterwards, the demand is almost constant.

We assume that the share of each of the demand components is independent and given by a specific distribution. The effect of competing zero-emission is considered by using scenarios with low hydrogen demand since some customer segments can decide for using battery electric solutions or ammonia instead of hydrogen. Since also the demand distribution is subject to uncertainty, we study the impact of different demand distributions on the infrastructure.

**Fig. 5:**
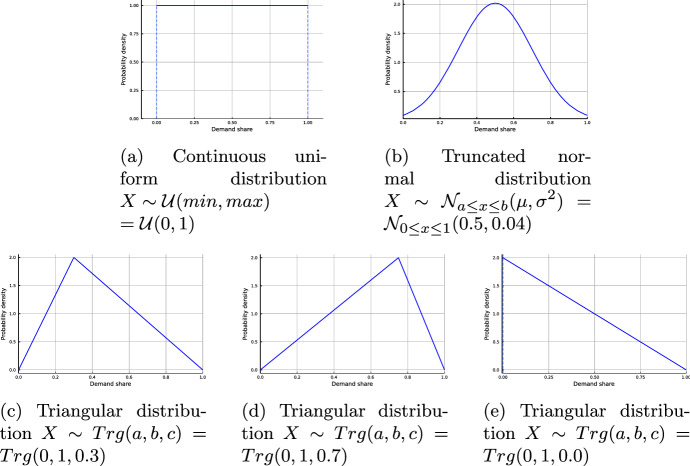
Probability density function of demand share distribution

To assess the impact the demand distribution has on the solution, we solve our model for a uniform (unif) distribution, a normal (norm) distribution, as well as three different triangular distributions, see Fig. 5. The uniform and the normal distributions have identical expected values, whereas the expected value of the triangular distributions depends on their shape. The left-skewed triangular distribution (trg-L) assumes demand to consist mainly of maritime demand and a low share of land-based and offshore demand, while the right-skewed triangular distribution (trg-R) assumes a high overall demand level. Finally, we present a left-skewed triangular distribution with the expected value equal to the maritime demand level with a very low of the Land-based and Offshore demand (trg-min).

We consider aggregated daily demand in 70 and 390 demand points located in Norway. For the maritime and offshore sectors, there are 51 demand points located in Norwegian ports. For the instances with 70 customers, we consider additional 19 municipalities with the highest road traffic volumes (Statistics Norway 2018). Road traffic demand is then divided among the 70 customers according to the relative traffic volume. For the instances with 390 demand points, we divide road traffic demand among the 390 municipalities. Note that municipalities with a daily hydrogen demand from road traffic of less than 10 kg are neglected.

## Computational results

All calculations have been carried out on a Linux cluster with two 3.6 GHz Intel Xeon Gold 6244 CPU (core) processors and 384 GB RAM. We use commercial software Gurobi Optimizer 9.5. to solve the demand allocation and facility utilization problem in our algorithm, as well as the LP relaxation of the problem and the original MIP to optimality. We implemented our algorithm in Julia 1.6.5. and enable parallelization on up to 32 threads.

We define the names of the problem instances by indicating the number of candidate facility locations (F), customers (D) and available capacity levels (C). For example, the problem instance F17D70C8 is a problem instance with 17 candidate facility locations, 70 customers and 8 available capacity levels. F17D70C8 also represents the real-world case of designing the hydrogen production infrastructure in Norway.

### Comparison with the expected value problem

**Fig. 6:**
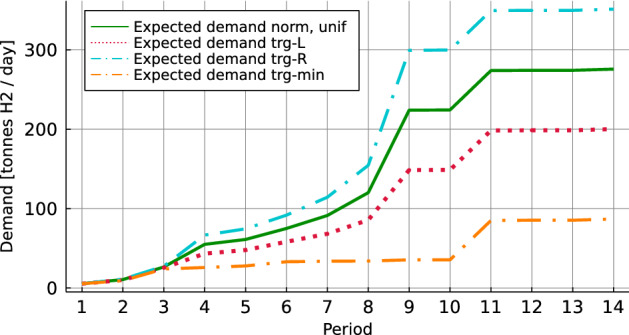
Expected demand level

For instance F17D70C8, we calculate the solution to the deterministic expected value problem (EVP) and compare the results with the stochastic problem (SP) using 3, 50, and 100 scenarios. We study the performance of first-stage solutions on a reference sample with 1000 scenarios for each distribution. When solving the EVP, the different scenarios are replaced by a single scenario where all customers request their expected demand. The expected demand level is illustrated in Fig. 6. Note that the normal and uniform distributions have identical expected demand and thus, identical EVPs, while each triangular distribution has different expected demand.

The expected value of the EVP solution (EEV) is calculated by evaluating the first-stage solution from the EVP over the reference sample with 1000 scenarios. We further evaluate first-stage decisions from solving the SP using 3, 50, and 100 scenarios over the reference sample with 1000 scenarios. The objective value is then denoted RP. Note that problems with 3 scenarios are solved to optimality using Gurobi (the Lagrangian heuristic provides solutions with a proven optimality gap $< 2\%$). The problems with 50 and 100 scenarios are solved with a proven optimality gap $< 4\%$ using the Lagrangian heuristic, while Gurobi cannot find any feasible solution within three days of computing time. The value of the stochastic solution (VSS) given as $VSS = EEV - RP$ (see, e.g., Birge and Louveaux 2011), provides a lower estimate of the true VSS since we do not solve the problem with 1000 scenarios to optimality.

**Table 4:** Out-of-sample evaluation for F17D70C8

dist	EEV $(10^6)$	3 scen		50 scen		100 scen	
		RP $(10^6)$	VSS $[\%]$	RP $(10^6)$	VSS $[\%]$	RP $[10^6]$	VSS $[\%]$
norm	37, 531.9	3, 134.3	91.65	2, 958.6	92.12	2, 945.5	92.15
unif	139, 380.4	3, 359.2	97.59	2, 935.5	97.89	2, 925.6	97.90
trg-L	2, 742.5	2, 726.8	0.57	2, 695.0	1.73	2, 692.1	1.84
trg-R	27, 339, 684.7	3, 702.1	99.99	3, 347.1	99.99	3, 279.6	99.99
trg-min	2, 565.8	2, 502.0	2.49	2, 318.6	9.63	2, 274.3	11.36

Results in Table 4 show the EEV and the RP considering first-stage solutions obtained for 3, 50, and 100 scenarios. For each RP solution, we calculate the relative VSS to EEV. The EVP solution for normal, uniform and right-skewed triangular distribution is feasible only when using penalties for capacity excess for scenarios with low demand. Both left-skewed triangular distributions (trg-L, trg-min) have sufficiently low expected values so that the penalties for capacity excess are avoided. Simultaneously, capacity expansion in the second stage provides sufficient flexibility to always avoid penalties for demand shortfall. This also applies if only maritime demand is considered when determining the locations and initial capacity of the production facilities to be opened (i.e., the first-stage decisions).

When increasing the number of scenarios from 50 to 100, first-stage decisions based on a solution to SP with 100 scenarios lead to lower RP. The improvement in RP when using 100 scenarios instead of 50 is at least $5.9\%$, $14.4\%$, $1.2\%$, $10.6\%$, $7.9\%$ for the normal, uniform and triangular distributions, respectively. Therefore, we focus on the results for 100 scenarios in our further evaluations.

**Fig. 7:**
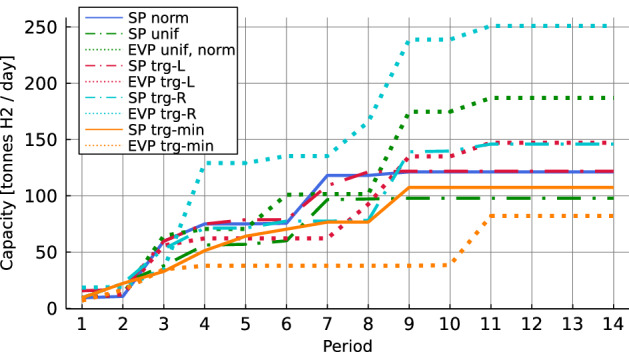
Installed capacity in the first stage (100 scenarios)

In general, the installed capacity in symmetric and right-skewed EVPs is considerably higher than the highest installed capacity among SPs (see Fig. 7) and therefore penalties for capacity excess apply in low-demand scenarios. The exceptions are the left-skewed distributions. The solution to EVP for trg-L installs slightly less capacity than the solution to SP in the first time periods while from period 9 onwards the installed capacity is higher. Note that in period 9, there is the most significant jump in the expected demand level for trg-L. The solution to SP leads to more conservative opening decisions to avoid low capacity utilization in scenarios where demand is realized below the expected value. Considering the distribution trg-min, the solution to SP installs more capacity to save expansion costs in scenarios where demand is realized above the expected value.

We can further observe (with exception of trg-min) that the installed capacity in SP is considerably lower than the expected demand while in the EVP, the installed capacity is close to the expected demand level. The reason is that capacity expansion is more expensive than opening a big facility right away. For a known demand level, the aim is to satisfy demand with very few expansions. Among the results for the SP, solutions for the uniform and the trg-min distributions lead to the lowest installed capacity. The uniform distribution is characterized by the highest variance among scenarios. Since the capacity level can be easily increased by expansion, the solution installs less capacity in the first stage to avoid low capacity utilization in scenarios with a low demand level.

When sampling multiple times, 3 scenarios are not always sufficient to avoid penalties even if the problem with 3 scenarios can be solved to optimality using Gurobi. In order to avoid penalties for capacity excess, at least one of the three scenarios has to be a scenario with a relatively low demand level which forces the solution to install less capacity. The solution to EEV for trg-min has shown that capacity expansion enables to increase the capacity, if necessary, and to avoid penalties for demand shortfall. Considering 50 and 100 scenarios, the probability of having a low-demand scenario in a sample is sufficiently large.

### Solution structure

**Fig. 8:**
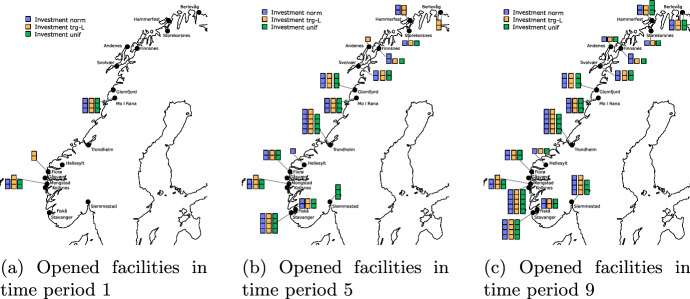
First-stage decisions: investment in the SP

To analyze the opening decisions for different demand distributions, we study the structure of the first-stage decisions for instance F17D70C8, solved with 100 scenarios with a proven optimality gap $< 4\%$. We focus on the normal, left-skewed triangular, and uniform distributions as these are considered to reflect plausible demand scenarios for Norway. The geographical locations of opened facilities in different time periods are shown in Fig. 8. We visualize the opened facilities in periods 1, 5, and 9, which allows us to analyze the main investment steps. Note that the solutions open the last new facility in period 9. From period 10 onwards, there are no additional first-stage opening decisions and demand increase is only compensated by capacity expansion.

In the first period (see Fig. 8a), the facilities are located in the middle of the southern and northern regions. These are strategic locations which can satisfy all customers without the necessity to open small local facilities. Surprisingly, the highest installed capacity is in the trg-L distribution, which is the distribution with the lowest expected demand. At the same time, this distribution is characterized by the lowest variance among scenarios. Therefore, the solution aims to install sufficient capacity to satisfy demand in more scenarios without expansion since opening right away a bigger facility is cheaper than expansion. These savings in investment costs compensate for higher production costs in low-demand scenarios with low capacity utilization. Figure 8b illustrates the opened facilities in period 5. We see that the solution opens most facilities when considering the trg-L distribution, while the fewest facilities are opened for the uniform distribution. The locations of the opened facilities are spread out along the entire coastline, irrespective of distribution. In period 9 (see Fig. 8c), 16 out of 17 possible facilities are opened. Hydrogen production is characterized by economies of scale. However, high distribution costs dominate economies of scale in production and therefore the solution chooses to open many relatively small facilities.

For all distributions, the largest facilities are opened in Kollsnes and Trondheim. Kollsnes has a strategic position on the west coast of Norway as most of the maritime customers and road traffic customers in the southern part of Norway are located within 1000 km distribution distance. Trondheim is an important location as it is the only location in the northern region that can supply road traffic customers in the southern part of Norway. A facility in Trondheim can therefore exploit both lower production costs due to lower electricity prices and economies of scale in production due to supplying municipalities with high demand.

Most of the production is located in the southern region. Even if production is cheaper in the northern region, distribution costs are high. If the distance travelled from a facility in the southern region is about 470 km shorter than from a facility in the northern region, it is favourable to use the facility located in the southern region. It would therefore be cheaper to supply all coastal customers south of Florø from local facilities rather than from Trondheim, even though the latter has cheaper production.

When considering a higher number of facilities, the number of opened facilities in the solution increases as well. Similar to the instance F17D70C8, due to high distribution costs, local production is preferred to centralized large-scale production in all instances. Further, most of the production is located in the southern region close to the customers. A facility located in the southern part of the northern region still plays plays a crucial role in supplying customers in the northern part of the southern region using the advantage of lower production prices. Further, a higher number of available capacities leads to lower objective. However, it does not lead to any structural changes in the infrastructure. The timing of the investment decisions is mostly affected by the demand curves and the fact that the production technology has minimum production requirements. Therefore, the opening of new facilities is in line with the demand increase. Since, the main characteristics of the solutions for larger instances are the same as we describe for instance F17D70C8, we have decided not to discuss them in detail.

### Solution quality

To analyze the quality of our lower bound, we compare it with the optimal solution to the MIP and with the LP relaxation bound and calculate the optimality gap. Given the complexity of the problem, Gurobi can find optimal solutions and solve the LP relaxation only for a few instances with 3 scenarios even when allowing four days of computing time. In Table 5, we provide the results for two different samples and demand distributions for the instance F17D70C8, the instance F25D70C16 for the left-skewed triangular distribution and the instance F17D70C16 for the normal distribution since Gurobi can find an optimal solution within four days of computing time only for these instances. When increasing the size of the problem size, neither the MIP nor the LP relaxation can be solved within the time limit. Table 5 shows the objective value of the LP relaxation and the Lagrangian bound as well as the time needed to solve the LP relaxation to optimality. Since the MIP optimal solution is known, we calculate the optimality gaps. Our Lagrangian heuristic finds good lower bounds within three hours with an optimality gap only slightly higher than the one of the LP relaxation (about $0.5\%$). For larger instances, the LP relaxation cannot be solved within four days which highlights the importance of scalable methods such as our Lagrangian heuristic.

**Table 5:** Quality of the bounds for 3 scenarios

Instance	dist	Gurobi		LR bound	Opt. gap $[\%]$	
		LP relax.		3h	LP	LR
		Obj ($\times 10^6)$	Time (s)	Obj ($\times 10^6)$	Relax	Bound
F17D70C8	norm	3563.4	1290	3561.7	0.47	0.52
F17D70C8	trg-L	2789.5	1378	2789.3	0.44	0.45
F17D70C8	unif	3658.1	936	3657.3	0.46	0.48
F17D70C8	norm	2892.7	1813	2891.1	0.41	0.47
F17D70C8	trg-L	3007.6	3022	3007.3	0.55	0.56
F17D70C8	unif	3198.0	1200	3196.7	0.33	0.37
F17D70C16	norm	2757.1	77473	2756.8	0.50	0.51
F17D70C16	trg-L	–	–	3311.5	–	–
F17D70C16	unif	–	–	3082.0	–	–
F25D70C16	norm	–	–	2836.3	–	–
F25D70C16	trg-L	3268.1	305954	3267.0	0.45	0.49
F25D70C16	unif	–	–	3136.3	–	–

**Table 6:** Computational results

Instance	dist	scen	1 h			5 h			Time to gap
			LB	UB	Gap	LB	UB	Gap	
			$(\times 10^6)$	$(\times 10^6)$	$[\%]$	$(\times 10^6)$	$(\times 10^6)$	$[\%]$	$< 5\%$
F17D70C8	norm	10	2972.2	3061.9	2.93	2972.7	3058.7	2.81	218
F17D70C8	trg-L	10	2868.4	2938.8	2.40	2868.9	2935.6	2.27	229
F17D70C8	unif	10	3135.3	3256.5	3.72	3135.8	3224.0	2.74	246
F17D70C8	norm	25	3065.4	3166.8	3.20	3065.4	3166.8	3.20	747
F17D70C8	trg-L	25	2730.6	2834.9	3.68	2731.0	2826.8	3.39	1331
F17D70C8	unif	25	2986.9	3121.6	4.32	2987.6	3089.3	3.29	589
F17D70C8	norm	50	2931.6	3041.3	3.61	2931.9	3041.3	3.60	3474
F17D70C8	trg-L	50	2762.6	2892.9	4.51	2763.0	2863.6	3.51	2433
F17D70C8	unif	50	2944.8	3089.2	4.68	2945.2	3064.7	3.90	1273
F17D70C8	norm	100	2949.4	3095.2	4.71	2950.3	3063.5	3.70	3043
F17D70C8	trg-L	100	2766.4	2895.5	4.46	2767.0	2877.5	3.84	2610
F17D70C8	unif	100	2905.9	3061.4	5.08	2906.7	3024.4	3.89	5099
F17D70C16	norm	100	2929.6	3063.1	4.36	2930.1	3009.8	2.65	2937
F17D70C16	trg-L	100	2749.1	2849.6	3.53	2749.5	2843.7	3.31	2610
F17D70C16	unif	100	2882.0	2988.3	3.56	2882.4	2982.9	3.37	2712
F25D70C8	norm	100	2908.7	3058.2	4.89	2909.0	3039.8	4.28	3248
F25D70C8	trg-L	100	2729.0	2848.6	4.21	2729.7	2848.8	4.18	3021
F25D70C8	unif	100	2861.6	3041.0	5.90	2862.7	2989.6	4.25	8654
F25D70C16	norm	100	2892.3	3058.3	5.43	2892.9	2981.2	2.96	3813
F25D70C16	trg-L	100	2713.3	2853.9	4.93	2714.0	2812.4	3.50	3516
F25D70C16	unif	100	2844.5	2991.2	4.91	2845.1	2940.3	3.24	3298
F34D70C8	norm	100	2903.6	3086.4	5.92	2903.9	3044.1	4.60	8807
F34D70C8	trg-L	100	2724.7	2847.9	4.32	2725.4	2847.9	4.30	2980
F34D70C8	unif	100	2856.4	3054.8	6.49	2857.5	2995.8	4.61	8295
F34D70C16	norm	100	2888.6	3077.7	6.15	2889.2	3007.9	3.95	5185
F34D70C16	trg-L	100	2710.0	2841.9	4.64	2710.5	2810.9	3.57	3532
F34D70C16	unif	100	2841.2	3018.0	5.86	2841.6	2960.7	4.02	6267
F17D390C8	norm	100	2787.8	3066.9	9.10	2814.0	2902.6	3.05	4373
F17D390C8	trg-L	100	2421.5	2618.8	7.53	2450.7	2548.5	3.83	4819
F17D390C8	unif	100	2669.1	2817.6	5.27	2695.7	2802.1	3.80	4068
F17D390C16	norm	100	2767.5	2997.2	7.66	2795.7	2867.9	2.52	5128
F17D390C16	trg-L	100	2396.8	2637.9	9.14	2433.5	2500.7	2.69	4474
F17D390C16	unif	100	2654.7	2919.9	9.08	2677.5	2754.7	2.80	3845
F25D390C8	norm	100	2727.5	3139.2	13.11	2779.0	2878.8	3.47	7727
F25D390C8	trg-L	100	2362.7	2734.2	13.58	2418.4	2515.8	3.87	10339
F25D390C8	unif	100	2611.9	2962.0	11.82	2662.5	2780.5	4.24	8531
F25D390C16	norm	100	2703.3	3027.8	10.72	2763.6	2857.0	3.27	6159
F25D390C16	trg-L	100	2337.4	2723.4	14.18	2403.6	2491.8	3.54	6326
F25D390C16	unif	100	2596.3	2997.7	13.39	2646.3	2734.1	3.21	5129
F34D390C8	norm	100	2697.1	3101.6	13.04	2772.2	2903.0	4.50	9855
F34D390C8	trg-L	100	2372.2	2631.1	9.84	2412.5	2526.2	4.50	6848
F34D390C8	unif	100	2581.4	3006.3	14.13	2627.1	2760.6	4.84	8951
F34D390C16	norm	100	2695.1	3009.3	10.44	2779.0	2878.9	3.47	7857
F34D390C16	trg-L	100	2315.3	2778.4	16.67	2400.5	2509.8	4.35	10645
F34D390C16	unif	100	2517.1	3098.3	18.76	2642.6	2761.1	4.29	8118

To discuss the performance of our algorithm and the quality of our solution, we show the results of our algorithm for 100 scenarios after 1 h and after 5 h of computing time in Table 6. We show the lower and upper bound, as well as the resulting gap and provide the computing time required to achieve a gap lower than $5\%$. For instance F17D70C8, we also show the results of our algorithm for 10, 25, 50, and 100 scenarios to assess the scalability of our algorithm when the number of scenarios increases.

Our Lagrangian heuristic finds good feasible solutions for all tested instances within a time limit of one hour. When increasing the time limit to 5 hours, we also observe a slight improvement in the lower bound. However, the main improvement is due to finding better feasible solutions with lower objective function values. After 5 hours, a solution with a proven optimality gap $< 5\%$ can be found for all tested instances.

The results show that for instances with a small number of scenarios, we find solutions with lower optimality gaps than for instances with a higher number of scenarios, since more iterations are performed and it is easier to find a first-stage solution that avoids penalties for all scenarios. However, the difference in solution quality between 50 scenarios and 100 scenarios is minimal.

Surprisingly, when increasing the problem size from 8 to 16 capacities, the resulting optimality gap tends to decrease, just as the run time needed to achieve a gap $< 5\%$. With 16 capacities, it is easier to find a suitable capacity level for the required production quantities than with 8. Therefore, our upper bound heuristic finds good solutions with low optimality gaps already in early iterations.

Since we allow for parallelization on up to 32 threads when calculating the lower bound, we further observe that increasing the number of candidate facility locations has a relatively low impact on the quality of our solution. However, with 34 candidate locations, we can see that the resulting gap increases as well as the time needed to achieve a gap $< 5\%$, since the number of iterations performed during the computing time decreases.

The instances with 390 customers are characterized by relatively long computing times to achieve an optimality gap $<5\%$. The time needed to update the Lagrangian multipliers increases as the size of the problem (29)–(33) depends on the number of customers and scenarios. In later iterations, updating the multipliers takes approximately $70\%$ of the time needed for one iteration. When increasing the time limit, we see a considerable improvement in the upper bound. However, the lower bound improves in average by $2\%$.

## Conclusion

We have studied the problem of locating hydrogen production facilities in Norway under demand uncertainty. We have formulated our problem as a two-stage stochastic multi-period facility location and capacity expansion problem considering minimum production requirements. The state-of-the-art commercial solver Gurobi can solve only the smallest instances with a low number of scenarios. Since the out-of-sample performance can be improved considerably when increasing the number of scenarios, we present a solution method based on Lagrangian relaxation to solve larger problems with a higher number of scenarios. With our algorithm, we find high-quality solutions for all tested instances within five hours computing time.

Results for small test instances indicate that our algorithm provides good lower bounds. Thus, for future work, the improvement potential lies within the upper bound heuristic. We further observed that the box-step method is a limiting factor for instances with a large number of customers, as the time needed to update the Lagrangian multipliers increases considerably. If shorter computing times are needed, exploring different methods or a combination of methods for the calculation of the Lagrangian dual may be a promising direction.

When solving our facility location model for the problem of locating hydrogen production in Norway, we see that due to high distribution costs, the solution chooses to open facilities at most of the candidate locations. Furthermore, most of the production is located in the southern part of Norway, since high distribution costs dominate the lower production costs in the northern part of Norway. The facility in Trondheim is therefore characterized by high opening capacity as it has low production costs and many road traffic customers in the southern part of Norway are within the distance limit. However, the demand scenarios used in our analysis are characterized by a large degree of uncertainty. We show that different distribution types do not have a large impact on size and location of the opened facilities. Still, more precise input data, in particular for future hydrogen demand, may provide a better basis for generating the scenario tree for our problem. Additional research efforts should therefore be dedicated to estimating future hydrogen demand, but this is outside the scope of the analysis in this paper.

The model can be further extended by including the choice of production technology. Together with including uncertainty in investment and production costs, the model might also be used to capture uncertainty in technology development. This is subject to future research.

## Data Availability

Not applicable
